# The Oral Microbiome Impacts the Link between Sugar Consumption and Caries: A Preliminary Study

**DOI:** 10.3390/nu14183693

**Published:** 2022-09-07

**Authors:** Liangyue Pang, Qinghui Zhi, Wenting Jian, Zhuoying Liu, Huancai Lin

**Affiliations:** Guangdong Provincial Key Laboratory of Stomatology, Department of Preventive Dentistry, Hospital of Stomatology, Guanghua School of Stomatology, Sun Yat-sen University, 56 Ling Yuan Road West, Guangzhou 510050, China

**Keywords:** oral microbiota, sugar consumption, dental caries

## Abstract

Background: The excessive and frequent intake of refined sugar leads to caries. However, the relationship between the amount of sugar intake and the risk of caries is not always consistent. Oral microbial profile and function may impact the link between them. This study aims to identify the plaque microbiota characteristics of caries subjects with low (CL) and high (CH) sugar consumption, and of caries-free subjects with low (FL) and high sugar (FH) consumption. Methods: A total of 40 adolescents were enrolled in the study, and supragingival plaque samples were collected and subjected to metagenomic analyses. The caries status, sugar consumption, and oral-health behaviors of the subjects were recorded. Results: The results indicate that the CL group showed a higher abundance of several cariogenic microorganisms *Lactobacillus*, *A. gerencseriae*, *A. dentails*, *S. mutans*, *C. albicans*, *S. wiggsiae* and *P. acidifaciens*. *C. gingivalis,* and *P. gingivalis,* which were enriched in the FH group. In terms of gene function, the phosphotransferase sugar uptake system, phosphotransferase system, and several two-component responses–regulator pairs were enriched in the CL group. Conclusion: Overall, our data suggest the existence of an increased cariogenic microbial community and sugar catabolism potential in the CL group, and a healthy microbial community in the FH group, which had self-stabilizing functional potential.

## 1. Introduction

Dental caries characterized by the demineralization of tooth tissue resulting from the fermentation of dietary carbohydrates by acid-producing bacteria [1]. The existence of cariogenic microorganisms is a prerequisite for the development of dental caries, and fermentable carbohydrates, particularly sugars, are key contributors to dysbiosis in the oral microbiota that promotes caries. Consuming sugar has negative effects on oral health, as cariogenic bacteria convert monosaccharides into acids that are detrimental to the teeth. Therefore, it is generally accepted that the excessive and frequent intake of refined sugar leads to the development of caries [2,3,4]. However, the relationship between the amount of sugar intake and caries risk is not always consistent [4,5]. In fact, some individuals with low sugar consumption still have a high caries risk, while some others are caries-free even with higher sugar consumption [4,5]. Many factors may influence the relationship between them, such as high socioeconomic status, exposure to fluoridated water, frequent tooth brushing, the availability of sugar for cariogenic bacterial digestion, and the availability of saliva to counteract bacteria and acids [5,6].

Previous studies suggested that oral ecological microbial profiles are corelated with the amount of sugar consumption [7]. Keller et al. found that some acidogenic and acid-tolerant caries-associated organisms were less abundant in a low-sugar group’s dental plaque [8]. Chen indicated that higher sugar-sweetened-beverage consumption may disrupt the oral microecology, and reduce the variety of microbiota during childhood, leading to an increase in cariogenic genera [9]. Altered plaque microbial profiles in adolescents with different levels of sugar intake were shown [8,9]. However, no study has explored the impact of oral microbiota on the relationship between sugar consumption and risk of caries.

We hypothesize that the oral microbiota of high-caries-risk adolescents with a low-sugar diet may have a special microbial community composition and function, leading to the formation of a local cariogenic environment with limited sugar. The microbial community of low-caries-risk subjects with higher sugar consumption may have some special bacterial antagonism to the cariogenic bacteria resulting in a state that protects teeth against caries even in a high-carbohydrate environment. Therefore, the purpose of this present study is to compare the oral microbial profiles of high-caries-risk adolescents with a low intake of free sugars to those of caries-free adolescents with a high consumption of free sugars.

## 2. Materials and Methods

### 2.1. Ethical Approval

This study was approved by the Ethical Committee of the Hospital of Stomatology, Sun Yat-sen University, in Guangzhou, China (KQEC-2021-24-03). Before this study, we obtained written informed consent from the parents or legal guardians of all participants.

### 2.2. Clinical Examination and Sampling

Prior to recruiting participants for the study, we performed an initial screening. During the initial screening, we conducted a structured questionnaire survey and clinical examination. The contents of the structured questionnaire were split into three parts: (1) Demographic information: sex, age, residence, and whether the child was an only child in their family and their primary caregiver. (2) Socioeconomic information: family income, caregivers’ educational levels, and whether they had dental insurance. (3) Oral-health-related behaviors: tooth-brushing frequency, flossing habits, toothpaste containing fluoride or not, mouthwash or not, frequency of snack consumption, frequency of sweet-drink consumption, and dental attendance experience. Caries status and plaque index were also evaluated. A skilled dentist used the International Caries Detection and Assessment System II (ICDAS-II) to measure the subjects’ caries experience, and recorded it as decaying, missing, and filled teeth (DMFT). The clinical examination was conducted under artificial lighting in the classroom by utilizing a dental mirror and a Community Periodontal Index (CPI) probe. Codes 3–6 in the ICDAS system were recorded as decayed teeth. Inclusion criteria were: healthy 12–13 years old adolescents who were caries-free and without any history of dental caries (DMFT = 0) or caries-active (DMFT ≥ 5) [10]. The plaque index of the Silness and Loe scale was used to record dental plaque. The subjects were excluded on the basis of the following criteria: (1): adolescents who had taken antibiotics during the past three months, (2) adolescents with bacterial or viral infections in other sections of the body, and (3) teenagers with gingivitis or wearing orthodontic appliances. In total, 40 adolescents with a similar average family income, composed of 20 caries-active adolescents and 20 caries-free adolescents, were enrolled.

Before the sample collection, breakfast was not permitted, and participating adolescents were not allowed to brush their teeth for 12 h. All the teenagers were instructed to rinse their mouths before the exam. Prior to sampling, the teeth were gently dried using an air stream to prevent saliva contamination. The buccal surfaces of the anterior and posterior teeth were scraped clean of dental plaque using a sterile scaler, and the plaque was then promptly transferred to a sterile 1.5 mL Eppendorf tube. The samples were brought to the laboratory as quickly as possible and frozen at −80 °C before analysis.

### 2.3. Questionnaire Survey and Free-Sugar Intake Assessment

The teenagers completed a questionnaire with three parts regarding their dietary habits and oral-health-related behaviors under the supervision of their parents. Part 1 was focused on the demographic information of students, Part 2 was primarily about students’ use of sugar-sweetened drinks (SSBs) and sweetened foods, and Part 3 was mostly concerned with oral health-related behaviors [11].

Part 1: gender, age, ethnic group, height and weight, residence, family income, caregivers’ education levels and whether they had dental insurance, whether the child was an only child in their family and their primary caregiver.

Part 2: included among SSBs were carbonated beverages, vegetable protein beverages, juice or juice drinks, tea beverages, sports beverages, and bubble tea. Cakes, desserts, candies (such as chocolate, Snickers, and Maltesers), and preserved fruits were all examples of foods that were sweetened (dried fruits and candied fruits). Other included as sources of free sugar were honey, flavored milk, and yogurt. For the typical intake of SSBs and sugary meals, the response options were 100, 200, 300, 400, and 500 mL/time, and 25, 50, 75, 100, 150, and 200 g/time.

Part 3: the frequency of tooth brushing, flossing habits, mouthwash or not, and whether the toothpaste contained fluoride.

The assessment of free-sugar intake referred to the study conducted by Q Lin et al. [11]. Following that, subjects were divided into two groups: those who consumed less than 50 g of sugar per day, and those who consumed more than 50 g of sugar per day.

### 2.4. Metagenomic Analysis

Each sample was subjected to the CTAB technique for the extraction of microbial DNA. The resulting pellet was redissolved into 50 L of TE buffer (10 mM Tris, 1 mM EDTA). The tube was filled with 1 μL of RNase A to digest the RNA, and it was then incubated at 37 °C for 15 min [12]. Nanodrop and agarose gel electrophoresis were used to evaluate the final DNA concentration and purity. The DNA libraries were created using the manufacturer’s instructions with the NEB Next^®^ Ultra TM DNA Library Prep Kit (Illumina, San Diego, CA, USA). The qualifying libraries were then sequenced at Wekemo Tech Co., Ltd. in Shenzhen, China using an Illumina NovaSeq PE150 platform. We handled the samples in accordance with the accepted procedures. During DNA extraction and library assembly, blank controls were used. In this analysis, there was no contamination in any of the samples. The collected raw reads were processed. Each sample was sequenced, and human reads were then removed from the metagenomic dataset by using Bowtie2 to align the DNA sequences to the human genome. Lastly, host-originated readings and low-quality reads were eliminated to produce clean data. The clean sequences of all samples were annotated and categorized, and Bracken was used to predict the actual relative abundance of species. We gathered data at seven taxonomical hierarchies from each sample using the algorithms of the lowest common ancestor [13] and gene abundance estimates [14].

Unsuccessful reads were filtered out after the unigene sequences had been aligned using DIAMOND (version 0.7.10.59, Benjamin Buchfink. Drost lab, Max Planck Institute for Biology, Tübingen, Germany) and the UniRef90 protein database. The gene abundance tables were obtained on the basis of the Uniref90 ID and the corresponding relationship of the KEGG database; then, the functional abundance profiles of each sample were drawn.

### 2.5. Data Visual Exhibition, and Statistical and Bioinformatic Analysis

The visuals and statistical analysis in this study were conducted using R software (version 3.8, Ross Ihaka and Robert Gentleman. The University of Auckland, Auckland, New Zealand) and QIIME (version 2, Rob Knight. University of California San Diego, San Diego, CA, USA). Fisher’s exact test was used to compare the clinical variables between groups. The Dunn test was used to detect significant differences in relative abundance between groups, with differences considered to be significant when *p* < 0.05. LEfSe analysis was used to compare microbial profiles and community functional structure, and the logarithmic LDA score threshold was set to 2.0. The core microbiome at the species level was depicted using a Venn diagram. Diversity analysis was carried out using nonmetric multidimensional scaling (NMDS) and principal coordinate analysis (PCoA) based on Bray–Curtis distance.

## 3. Results

In total, 40 male and female teenagers aged 12–13 years were recruited; 45% were boys, and 55% were girls. The samples were divided into four groups: caries individuals with low sugar consumption (CL group), caries individuals with high sugar consumption (CH group), caries-free individuals with low sugar consumption (FL group), and caries-free individuals with high sugar consumption (FH group). Table 1 reports the characteristics of the demographic variables and the oral hygiene habits of the included subjects. As presented in Table 1, there was no difference in the clinical information that could be confounding variables among the four groups.

**Figure 1:**
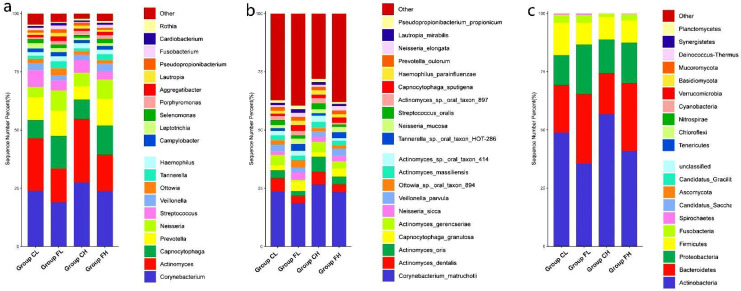
Relative abundance of the 20 most predominant (**a**) phyla, (**b**) genera, and (**c**) species among groups.

After data trimming and filtering out the low-quality data and host contamination, a total of 651,389,975 (195.43 GB) reads were acquired, and the average clean data were 4.89 GB per sample. A total of 22 phyla, 36 classes, 93 orders, 214 families, 761 genera, and 2235 species were detected in the 40 samples. Figure 1a–c shows the top 20 phyla, genera, and species, respectively, with relatively high abundance in the four groups.

### 3.1. Microbiota Composition Based on Sugar Consumption and Caries Status

**Figure 2:**
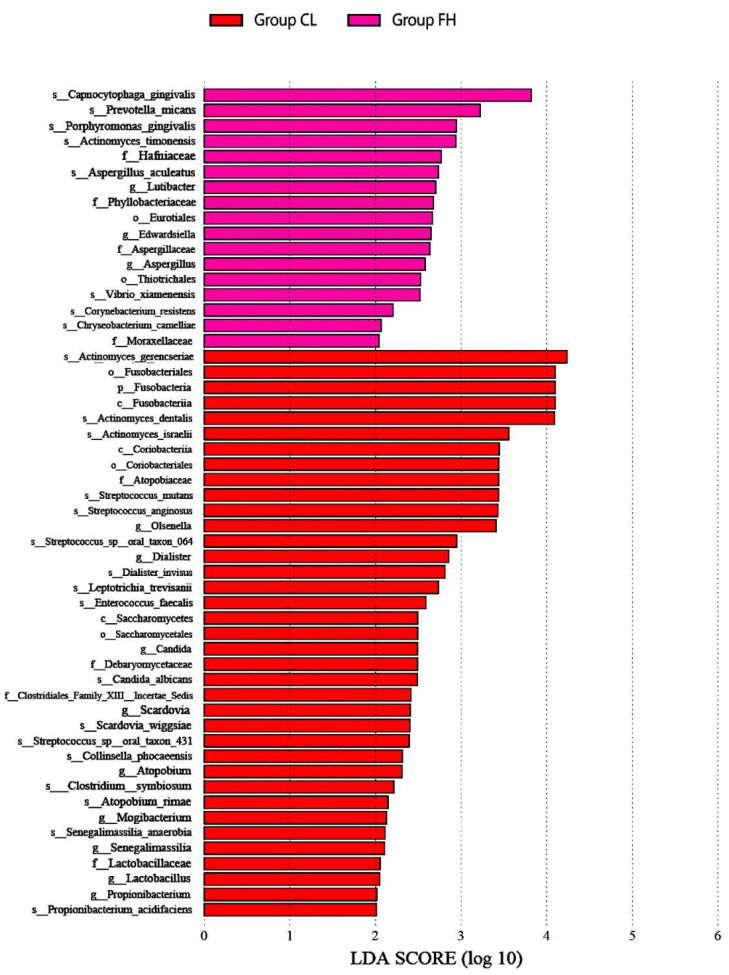
Relative abundance of phyla and species that were significantly higher in the FH and CL groups compared with the three other groups in LEfSe analysis (Dunn test, *p* < 0.05).

The bacterial distribution within the different groups was analyzed. We compared the relative abundance of the top 20 bacteria in the four groups with some interesting findings. Figure 2 shows the relative abundance of the phyla and species that were significantly higher in the FH and CL groups compared with the three other groups. As illustrated in Figure 2, at the phylum level, the CL group showed a higher relative abundance of Fusobacteria. At the genus level, the CL group was associated with a greater abundance of *Lactobacillus*. At the species level, in the CL group, *Actinomyces gerencseriae* and *Actinomyces dentails* had a higher relative abundance of the top 20 species covered. *Streptococcus mutans*, *Candida albicans*, *Scardovia wiggsiae,* and *Propionibacterium acidifaciens* were also more abundant in the CL group out of the top 20 species covered. However, eight species were more abundant in the FH group, including *Capnocytophaga gingivalis* and *Porphyromonas gingivalis* (the figure titled “Significant differences in relative abundance of plaque microbial taxa among the four groups by LEfSe analysis” was too large to place in the manuscript, so we were uploaded it as Supplemental Material Figure S2).

**Figure 3:**
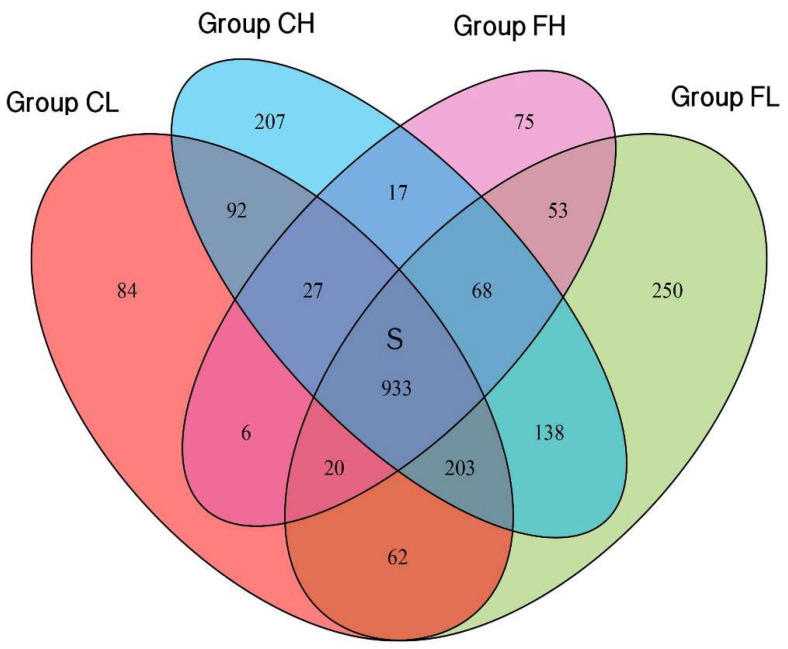
Venn diagram at species level.

Venn diagram was used to illustrate the shared and unique taxa for the species level among groups. A total of 2235 species were identified in our study. The overlap region S indicates the microbiome shared in all samples among the four groups, and 933 species were detected in all of the four groups. There were 84, 207, 75 and 250 unique species detected in the CL, CH, FH, and FL groups, respectively (Figure 3).

**Figure 4:**
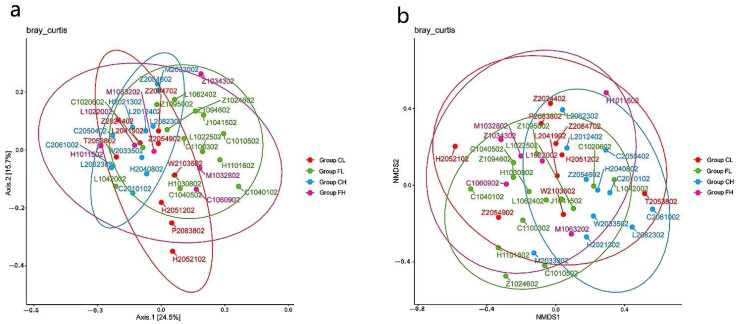
Diversity analysis between groups: (**a**) PCoA and (**b**) nonmetric multidimensional analysis based on Bray–Curtis distances.

PCoA (Figure 4a) and NMDS (Figure 4b) did not reveal differences in taxonomic unit structural richness among groups. The results demonstrate that the microbial diversity of the four groups was similar.

### 3.2. Microbiota Composition Based on Sugar Consumption and Caries Status

**Figure 5:**
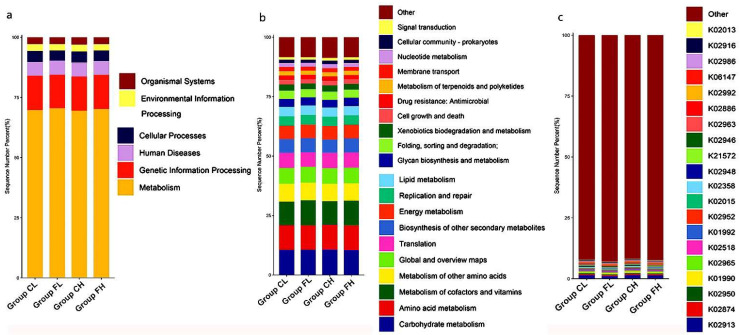
Relative abundance of the 20 most predominant annotated of functional KEGG between groups at (**a**) Level 1 pathway, (**b**) Level 2 pathway, and (**c**) ortholog.

We annotated the oral gene catalogs with the Kyoto Encyclopedia of Genes and Genomes (KEGG) database in order to compare the functional characteristics of the microbiota between groups. The metabolic pathway that includes 5923 annotated genes was the most abundant of the six main KEGG pathways that were found (Figure 5a). There were 47 Level 2 pathways, with the metabolism of the carbohydrates being the most abundant (Figure 5b). The metabolism of cofactors and vitamins, and amino acid metabolism were other common markers in the Level 2 pathways. The top 10 most abundant KOs were discovered by performing BLAST against the KEGG Orthology (KO) database, and they are shown in Figure 5c.

**Figure 6:**
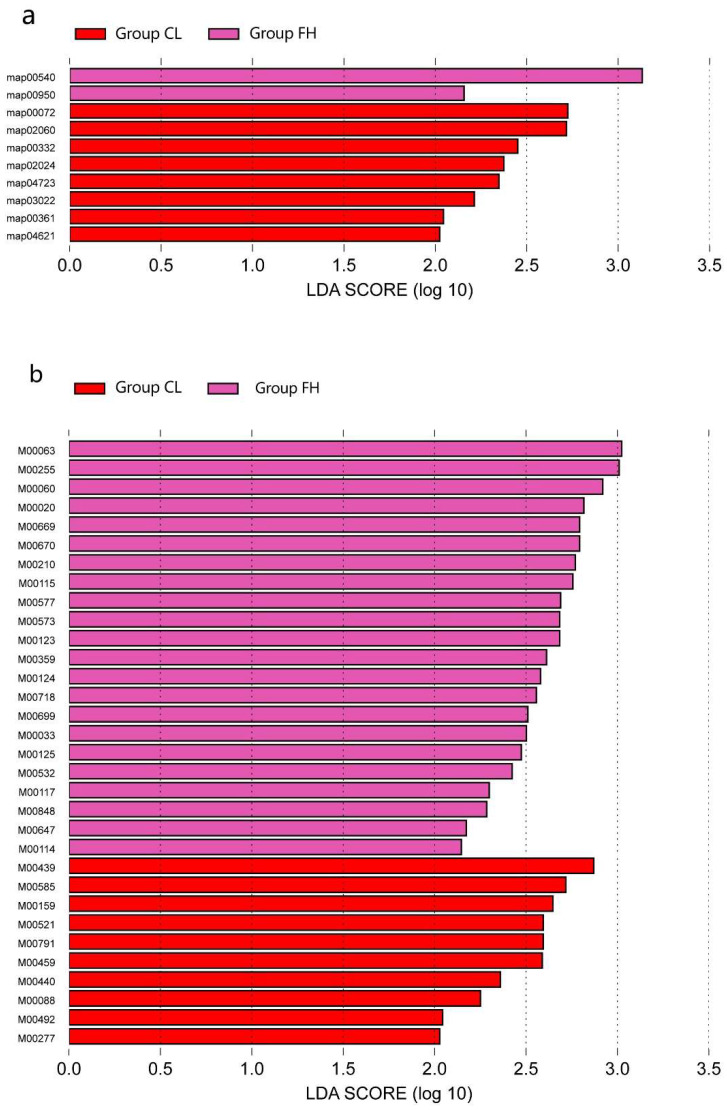
LEfSe analysis was used to identify level 3 (**a**) and Module level (**b**) with significant differences in relative abundance between groups and only the different maps and modules in the CL group were shown in the figure.

LEfSe analysis of the gene profiles showed enormous differences at the functional level of the groups. At the second level, enrichment in membrane transport was observed in the CL group compared with the other groups. At the third level, the synthesis and degradation of ketone bodies, the phosphotransferase system (PTS), carbapenem biosynthesis, quorum sensing, retrograde endocannabinoid signaling, basal transcription factors, chlorocyclohexane and chlorobenzene degradation, and the NOD-like receptor signaling pathway were enriched in the CL group, while lipopolysaccharide biosynthesis and isoquinoline alkaloid biosynthesis exhibited a higher level in the FH group (Figure 6a).

In total, 10 KEGG metabolic modules were positively associated with the CL group’s microbiomes, while 22 KEGG metabolic modules had a higher proportion in the FH group (Figure 6b). Phosphotransferase sugar uptake system N-acetylgalactosamine (M00277), numerous two-component histidine kinase-response regulator systems including VicK/VicR (cell wall metabolism) (M00459), LytS-LytR (M00492), and CiaH/CiaR (M00521), and several trace metal transport systems, including those for manganese/zinc (M00791) and nickel (M00440), were more enriched in the CL group. Modules associated with biosynthesis were more abundant in the FH group, including serine (M00020), ectoine (M00033), lipopolysaccharide (M00060), CMP-KDO (M00063), ascorbate (M00114), NAD (M00115), ubiquinone (M00117), biotin (M00123), pyridoxal (M00124), Riboflavin (M00125), aminoacyl-tRNA (M00359), biotin (M00573, M00577), and aurachin (M00848). The FH group also had enriched xenobiotic efflux pump transporters, including multidrug resistance, AcrAB-TolC/SmeDEF (M00647), multidrug resistance, AmeABC (M00699), multidrug resistance, MexAB-OprM (M00718) (the figure titled “LEfSe analysis was used to identify Level 3 and module level with significant differences in relative abundance among the four groups” was too large to place in the manuscript, so we uploaded it as Supplementary Figure S2).

## 4. Discussion

The present study employed metagenomic sequencing to characterize the tooth plaque microbiota in a group of Chinese adolescents with a focus on microbiomic profiles and function in caries subjects with low sugar consumption and caries-free subjects with high sugar consumption. The main findings were that oral microbial profiles and function impact the relationship between sugar consumption and caries.

For this pilot study, we compared the microbial profiles of the subjects with comparable backgrounds. Since all the adolescents resided in the same city (Foshan), there were no racial, cultural, demographic, or geographical distinctions in the selected sample. Examined individuals had an average monthly family income between CNY 3000 and 7000, so it was reasonable to assume that their children had a comparable socioeconomic status. In addition, the water in Foshan has not been fluoridated. Therefore, the individuals’ fluoride intake comes from fluoride-containing toothpaste and mouthwash. Since none of the individuals used mouthwash, the only available source of fluoride for the subjects was fluoride toothpaste. We found no statistically significant difference among the four groups in their use of fluoride toothpaste. The same held true for oral hygiene habits, the plaque index, the pH value of saliva, and the buffering capacity of saliva. We, therefore, presumed that all children had comparable backgrounds.

In high caries risk subjects with a low sugar consumption, the relative abundance of Lactobacillus was increased, as well as the relative abundance of several species including *Actinomyces dentails*, *Actinomyces gerencseriae*, *Streptococcus mutans,* and *Candida albicans*. These species are frequently associated with caries progression [15]. Consistent with previous studies, it is not surprising to find a high relative abundance of *Lactobacillus* in the CL group [16]. Before *S. mutans* was found, it had been thought that *Lactobacillus* was the main cause of caries because there was a strong association between the amount of Lactobacillus in saliva and the severity of caries [5]. *Lactobacillus* is currently thought to not be the caries initiator, but that it plays an important role in caries progression [17].

These findings suggest that even individuals with low sugar consumption may have a high caries risk when the abundance of some cariogenic bacteria, such as *Actinomyces gerencseriae*, *Scardovia wiggsiae*, *Propionibacterium acidifaciens*, *Streptococcus mutans,* and *Candida albicans,* is increased. Clinical studies linked high levels of *Streptococcus mutans* (*S. mutans*) to high caries risk due to its acidogenicity, acidification, and ability to synthesize exopolysaccharides in dental plaques [12]. *Candida albicans*, a commensal oral fungus, can form pathogenic mixed-species biofilms with *S. mutans* mediated by extracellular polysaccharides (EPSs), enhancing the virulence leading to the onset of caries. Previous studies confirmed that, when *C. albicans* is combined with *S. mutans* in a rat model, it causes more severe dental caries [18,19], and coinfection with *S. mutans* and *C. albicans* is highly related with severe early child caries [20,21]. The present findings further support this result, indicating that the increased abundance of *S. mutans* and *C.albicans* is closely related to the increased risk of caries.

*Scardovia wiggsiae* (*S. wiggsiae*) was described as a new caries pathogen that was detected from caries in children and adolescents [22,23]. *S. wiggsiae* showed acid tolerance and acidogenicity, increasing its ecological competitiveness in acidic conditions such as caries lesions [24]. *S. wiggsiae* exhibited minimal caries induction by itself. However, when co-inoculated with *S. mutans*, significant cavity formation was observed [25], and Eriksson et al. found that adolescents with active caries were characterized by a bacterial complex that contained *S. mutans* and *S. wiggsiae* [23]. In agreement with previous studies, we also observed higher relative abundance of *S. wiggsiae* in the CL groups as compared with the three other groups. Other bacteria found in our study to be more abundant in the CL group were *Actinomyces gerencseriae* and *Propionibacterium acidifaciens*. These two bacteria were positively associated with caries [26,27]. Similar findings were described in our previous study with pit and fissure caries [14].

In our study, the FH group had a higher abundance of *Capnocytophaga gingivalis* (*C. gingivalis*) and *Porphyromonas gingivalis*. Consistent with our findings, *C. gingivalis* is frequently detected in subgingival plaque associated with oral health [16]. *Porphyromonas gingivalis* (*P. gingivalis*) is a common periodontal pathogen that appears in greater proportion in periodontitis than it does in healthy subjects [28]. Additionally, *P. gingivalis* salivary levels in a caries-free group were considerably greater than those in the periodontally healthy group with caries, according to Y Iwano [29]. A possible explanation is that *P. gingivalis* is antagonistic to *S. mutans* [30]. The former can attenuate the virulence properties of the latter by interfering with its Com quorum-sensing system [31]. As a result, FH subjects should have a healthy microbial community capable of resisting the cariogenic microbial population.

The result of the functional analyses indicates significant differences among groups at various functional levels. A quorum-sensing system is a cell-to-cell signaling system allowing for bacteria to develop coordinated social behavior [32]. The quorum-sensing system exhibited a higher level in the CL group, suggesting active signaling inside the biofilm. We also found an enrichment of the phosphotransferase sugar-uptake and phosphotransferase systems in the CL group, indicating enrichment for the sugar-uptake, transport, and metabolization pathways. Moreover, a notable enrichment of several two-component response–regulator pairs that are responsible for transcriptional changes in reaction to environmental stimuli was observed in the CL group. Relman DA pioneered the theory that increases in sugar catabolism potential are key markers of caries development and progression [33]. According to our research results and those of previous studies, we suggest the existence of a cariogenic microbial interaction network. We speculate that sugar uptake, transport, and metabolization capacity, and the response to the environmental stimuli of the microbial community increased in CL group subjects. Therefore, in the presence of limited sugar, the cariogenic microbial community of the CL group could respond quickly and metabolize sugar to rapidly produce acid, leading to a low-pH microenvironment within the biofilm. Additionally, the microbial community of FH subjects had self-stabilizing functional potential in which sugar compounds were not easily taken up by the microorganism, preventing the associated pH decrease.

## 5. Conclusions

In conclusion, findings from both previous studies and the current study lend indirect support to the notion that the caries phenotype is a disorder of the microbial community metabolism. In the CL group, even with a lower sugar intake, the subjects had a high caries risk because of the existence of a cariogenic microbial community and increased sugar catabolism potential. Even though the subjects in the FH group take up more sugar, they had a low caries risk due to a healthy microbial community being capable of resisting the cariogenic microbial population and preventing pH reduction by inhibiting sugar uptake. The present study has the obvious shortcoming of a limited sample size that only permits preliminary findings. In addition, sugar consumption data were collected via questionnaire, and some free sugars were not included, such as sucrose in homemade dishes. Given this, it is prudent not to overstate the consequences of the findings. Future investigations on a larger scale are required to confirm and validate our findings.

## Figures and Tables

**Table 1:** Comparison of demographic variables and oral hygiene habits between groups.

Characteristics	CL (*n* = 9)	FL (*n* = 14)	CH (*n* = 11)	FH (*n* = 6)	*p*
Frequency of tooth brushing					0.75
<2 time a day	4	9	7	3	
≥2 time a day	5	5	4	3	
Toothpaste containing fluoride or not					0.12
Yes	8	10	11	6	
No	1	4	0	0	
Floss or not					0.32
Yes	0	3	2	0	
No	9	11	9	6	
Plaque index					0.91
1–2	5	9	7	3	
0–1	4	5	4	3	
BMI status (kg/m^2^)	16.96 ± 1.29	18.86 ± 2.4	19.69 ± 5.9	17.44 ± 1.26	
Free sugar consumption (g/day)	27.28 ± 20.67	33.85 ± 23.33	221.77 ± 113.87	246.74 ± 261.83	

## Data Availability

Not applicable.

## Supplementary Materials

The following supporting information can be downloaded at: https://www.mdpi.com/article/10.3390/nu14183693/s1, Figure S1: Significant differences in relative abundance of plaque microbial taxa among groups by LEfSe analysis. Figure S2: LefSe analysis was used to identify Level 3 and module level with significant differences in relative abundance among groups.
