# Supplementary figures and images for: The Oral Microbiome Impacts the Link between Sugar Consumption and Caries: A Preliminary Study
Source: Nutrients. 2022 Sep 7;14(18):3693. doi: 10.3390/nu14183693 (PMC9503897; doi:10.3390/nu14183693)

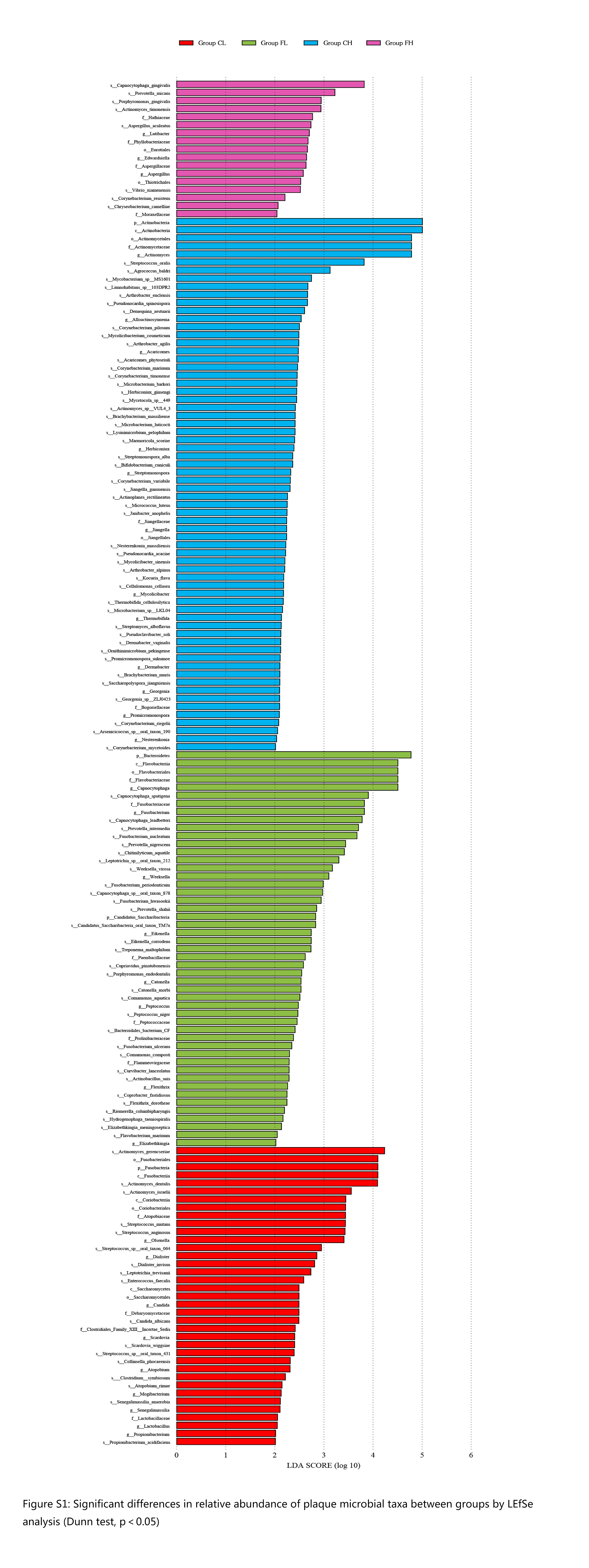

Supplement: Supplementary file 1 [file nutrients-14-03693-s001.zip › nutrients-1846287-supplementary/Figure S1.jpg]

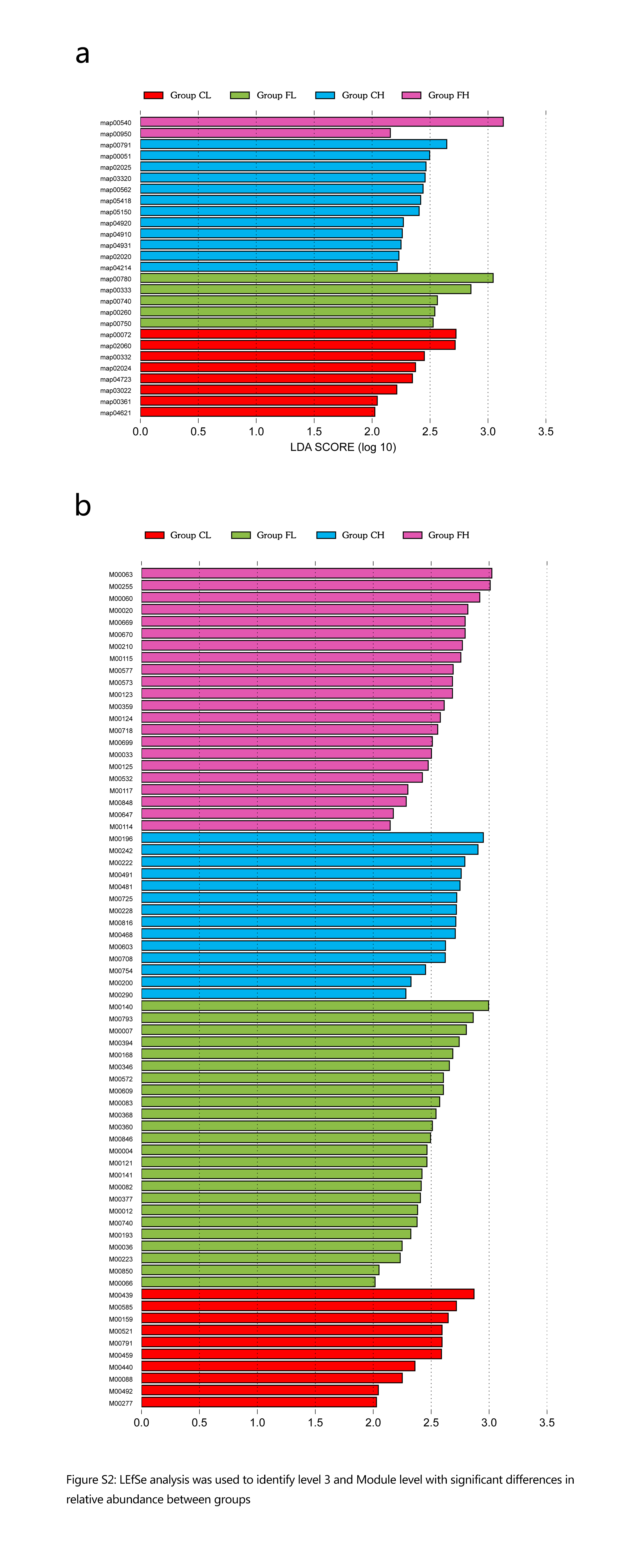

Supplement: Supplementary file 1 [file nutrients-14-03693-s001.zip › nutrients-1846287-supplementary/Figure S2.jpg]
